# Advances and innovations in ultrasound-based tumor management: current applications and emerging directions

**DOI:** 10.1186/s13089-025-00444-2

**Published:** 2025-08-12

**Authors:** Li Rui, Qi Min, Meng Xin, Chen Yujun, Gu Yihong, Yan Ruilong, Wang Bo, Yu Tengfei

**Affiliations:** 1 Department of Ultrasound, Beijing Tiantan Hospital, Capital Medical University, Beijing, China; 2 Department of Vascular Ultrasonography, Xuanwu Hospital, Capital Medical University, Beijing, China; 3 Department of Radiology, Qingdao Municipal Hospital, Qingdao Hospital, University of Health and Rehabilitation Sciences, Qingdao, Shandong China; 4 Department of Ultrasound, Qingdao Eighth People’s Hospital, Shandong Second Medical University, Shandong, China; 5 Guangdong Key Laboratory of Biomedical Measurements and Ultrasound Imaging, School of Biomedical Engineering, Shenzhen University Medical School, Shenzhen University, Shenzhen, China

**Keywords:** Ultrasound, Tumor, Molecular imaging, SDT, Thermal therapy, Immunoregulation

## Abstract

As a crucial medical imaging modality, ultrasonography has emerged as a pivotal tool for tumor diagnosis and treatment owing to its non-invasive nature, real-time imaging capability, and superior resolution. Recent technological advancements have demonstrated unique advantages in early tumor screening, staging, and localization. Contrast-enhanced ultrasound (CEUS), utilizing microbubbles (MBs) and nanobubbles (NBs) to target vascular biomarkers, significantly enhances tumor visualization and demonstrates high sensitivity in molecular imaging. Multimodal ultrasound (MU), incorporating techniques such as elastography and automated breast volume scanning (ABVS), achieves improved diagnostic accuracy when combined with MRI/CT. The applications of ultrasound in localized and systemic tumor therapy have expanded considerably. High-intensity focused ultrasound (HIFU) enables thermal ablation of solid tumors, while low-intensity focused ultrasound (LIFU) facilitates sonodynamic therapy (SDT) through reactive oxygen species (ROS) generation mediated by sonosensitizers. Ultrasound-assisted drug delivery systems (US-DDS) leverage MB/NB cavitation effects to enhance chemotherapeutic agent delivery efficiency, overcome biological barriers, including the blood-brain barrier, and modulate immune responses. These technological breakthroughs have provided novel therapeutic options for cancer patients, garnering significant clinical interest. This review systematically examines current applications of ultrasound imaging and therapy in oncology, evaluates its potential clinical value, analyzes existing technical limitations, and discusses future development prospects. The article aims to provide innovative perspectives for tumor diagnosis and treatment while offering references for clinical practice.

## Introduction

**Fig. 1:**
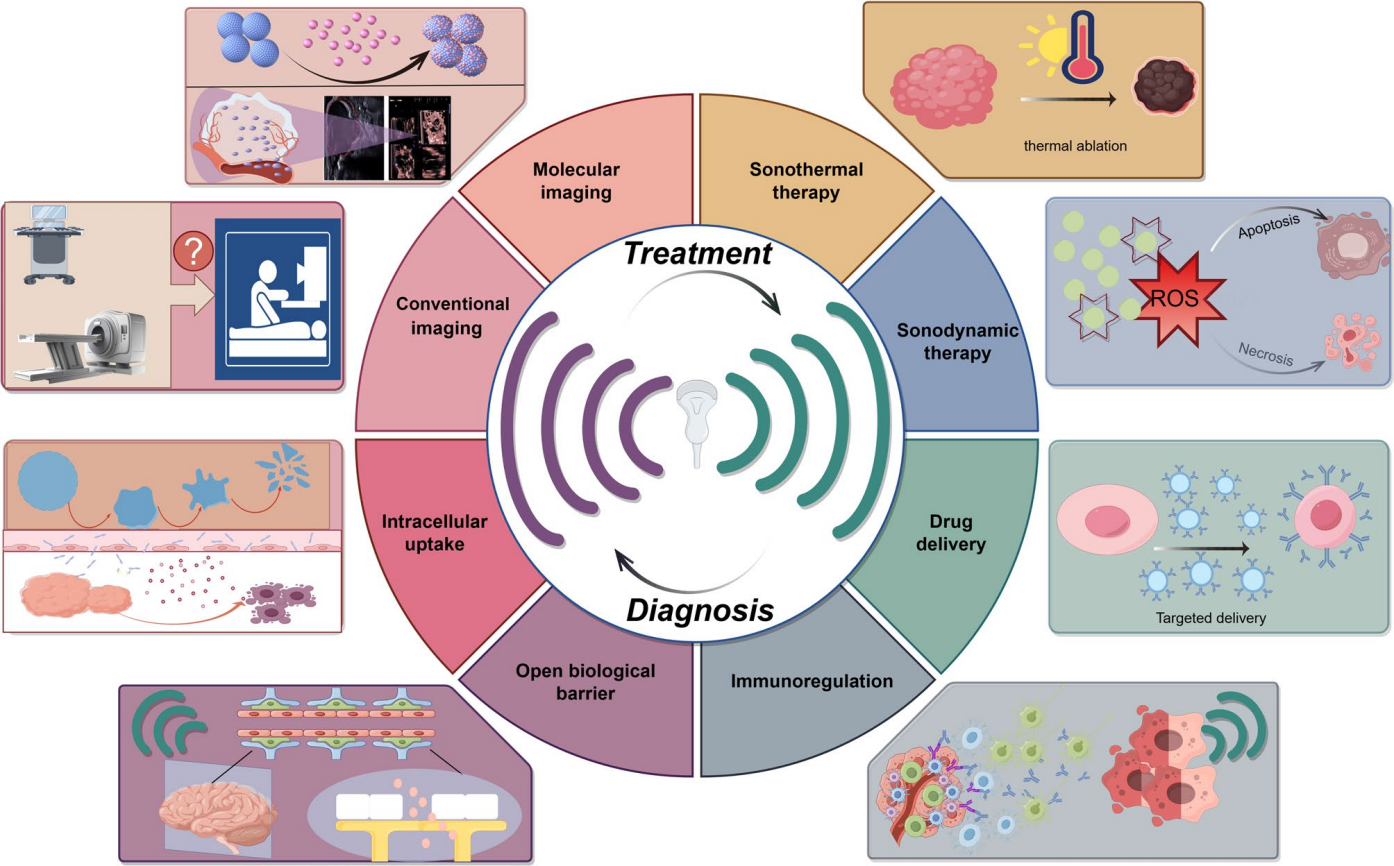
Role of Ultrasound in Tumor Management

Globally, tumors represent a major public health concern due to their rising incidence and high mortality rates. Recent epidemiological data indicate that tumors have become the second leading cause of death worldwide, significantly affecting patients’ quality of life and imposing considerable social and economic burdens on healthcare systems. Consequently, there is a critical need for effective strategies in early screening, accurate diagnosis, and advanced therapeutic approaches to improve patient prognosis and survival rates [1, 2]. In recent years, advancements in ultrasound (US) technology have significantly expanded its role in tumor management, ranging from diagnostic imaging to complex interventional procedures. US offers real-time, non-invasive imaging with high sensitivity and specificity, making it a widely adopted tool in clinical practice. It enables precise localization of tumors during biopsies and interventional treatments. Moreover, the therapeutic use of US has steadily increased, including US-guided tumor localization, ablation, and the targeted delivery of chemotherapeutic agents, thereby providing innovative treatment options for cancer patients. This article aims to provide a reference for current clinical applications and explore future advancements in US technology to enhance its value in tumor diagnosis and treatment (Fig. 1).

## Application of US imaging in tumor diagnosis

### Basic principles of US imaging technology and clinical applications

US refers to sound waves with frequencies above the human hearing threshold (greater than 20 kHz) and is characterized by high resolution, deep tissue penetration, efficient energy conversion, and rapid propagation speed. US imaging leverages these properties by emitting high-frequency sound waves and capturing their echoes to generate images. As these waves travel through tissues with different densities and elasticities, variations in propagation speed and reflection intensity occur. The US probe detects the reflected signals and converts them into electrical impulses, which are then processed by a computer to produce visual images commonly used in clinical diagnostics [3].

Early diagnosis and accurate tumor staging are critical for improving treatment outcomes and patient survival rates. Conventional imaging techniques such as magnetic resonance imaging (MRI), computed tomography (CT), and X-rays play essential roles in tumor identification and staging. However, each of these modalities has inherent limitations, including exposure to ionizing radiation, prolonged imaging times, and inadequate resolution for certain tumor tissues. As a result, the search for safer and more efficient imaging methods has become a major focus of research. US has gained increasing prominence in tumor monitoring and early screening due to its non-invasive nature, real-time imaging capabilities, and portability [4]. Multimodal US (MU), which integrates various US technologies, has emerged as a powerful tool for enhancing the accuracy of tumor detection and treatment planning. Recent advances in US technology have elevated MU to a pivotal role in tumor diagnostics, improving tumor visualization and providing critical information during real-time monitoring to support clinical decision-making [5]. For instance, MU demonstrated a sensitivity of 97.85% in diagnosing benign and malignant liver tumors, compared to 82.56%, 92.39%, and 87.14% for single US, contrast-enhanced US (CEUS), and shear wave elastography (SWE), respectively [6]. In another study, Ma et al. developed a MUmodel combining an automated breast volume scanner (ABVS) and strain elastography (SE) with B-mode US features, which enhanced the differentiation between benign and malignant breast tumors [7]. Furthermore, multimodal imaging that combines US with MRI and CT has shown significant advantages in tumor diagnosis. For example, in the evaluation of suspected ovarian tumors, US serves as the first-line imaging modality. However, MRI is essential as a second-line tool for characterizing indeterminate adnexal masses, given the overlapping US features of various ovarian lesions. When either US or MRI raises suspicion of ovarian cancer, the International Federation of Gynecology and Obstetrics recommends using CECT to assess disease extent. This imaging strategy provides clinically relevant information such as the degree of primary tumor spread, presence of peritoneal implants, and the size and location of lymph nodes—all crucial for treatment planning and assessing the feasibility of cytoreductive surgery [8]. Therefore, the combined use of US, CT, and MRI enhances diagnostic accuracy and reliability throughout the various stages of tumor assessment.

### The role of CEUS in tumors

Technological advancements have enabled US imaging to support multimodal, comprehensive assessments across a wide range of tissues and organs, highlighting its substantial potential for the early screening of specific tumor types. CEUS is a diagnostic technique that involves the intravenous injection of US contrast agents (UCAs), followed by the emission of sound waves from the US transducer. UCAs produce nonlinear acoustic responses, whereas surrounding tissues primarily generate linear signals. This difference enhances tissue contrast by suppressing the linear components from the tissue background and amplifying the nonlinear signals from the contrast agents [2]. CEUS is based on the evaluation of microvascular architecture and the relative contrast enhancement of the target lesion compared to adjacent healthy tissues, thereby assisting clinicians in characterizing tumors and determining their stage [9].

#### CEUS based on microbubbles (MBs)

Lipid-shelled, gas-filled MBs are the most commonly used UCAs in clinical practice to enhance the image quality of CEUS. Several commercially available UCAs—such as Levovist, Definity, Optison, Sonazoid, and SonoVue—have received approval for clinical use by the U.S. Food and drug administration (FDA) [10]. Previous studies have demonstrated that CEUS can effectively differentiate between benign and malignant ovarian tumors, achieving sensitivities and specificities of 90% and 85%, respectively [11]. Additionally, CEendoscopic US has shown high sensitivity in evaluating the microvascular density of pancreatic tumors, where reduced vascular density may indicate greater tumor aggressiveness in patients with non-functional pancreatic neuroendocrine tumors [12]. Notably, Cui et al. successfully identified sentinel lymph nodes (SLNs) in early breast cancer patients using Sonazoid, reporting a detection rate of 100%, with a sensitivity of 92.31% for identifying non-involved SLNs and a negative predictive value of 96.79% [13]. The early diagnosis of lung cancer remains clinically challenging, particularly for small nodules or early-stage lesions. CEUS is gaining recognition for its utility in lung cancer diagnosis, as it can enhance visualization of blood flow within lesions, assess vascular supply, determine the extent of tumor infiltration, and evaluate spatial relationships with surrounding tissues [14, 15]. Moreover, CEUS can guide puncture biopsies of lung lesions, thereby increasing sampling accuracy and success rates [16]. In a study involving 127 patients with ductal carcinoma in situ (DCIS), CEUS significantly improved lesion detection by providing clearer lesion boundaries and more detailed blood flow characteristics [13]. Progress has also been made in the development of targeted MBs for CEUS. For example, Hu et al. designed a novel vascular-targeted contrast agent, B7-H3, aimed at preventing breast cancer metastasis and facilitating targeted SLN removal [17]. In a mouse model, they tested the agent using 21 MHz CEUS to image both metastatic and non-metastatic SLNs, yielding stronger imaging signals. However, the continued advancement of CEUS remains limited by intrinsic challenges associated with MBs, including short lifespan, low stability, and size heterogeneity [18].

#### US molecular imaging

MBs, typically ranging in size from 1 to 10 μm, are limited in their imaging applications to the vascular system due to constraints such as short circulation time and limited structural control [19, 20]. In contrast, US contrast agents with smaller diameters offer improved tissue penetration and can overcome these limitations [21, 22]. Nanobubbles (NBs), also referred to as submicron or nanoscale bubbles, are present in some commercial microbubble formulations, such as Definity^®^. The development of US NBs was initiated by Wheatley et al. in 2004, with their first in vivo activity demonstrated in 2006 [23, 24]. Since then, the application of NBs as contrast agents in biomedical ultrasonography has gained increasing recognition. Notably, Rapoport et al. reported the selective imaging of tumor stroma using NBs [25]. However, widespread use of US NBs did not begin until around 2010, when their potential in tumor imaging began to be highlighted in several studies [26–29]. More recently, US molecular imaging has emerged as a prominent research focus within the context of CEUS. This approach involves the construction of targeted acoustic contrast agents by conjugating specific antibodies or ligands to the surface of the contrast agent, enabling active binding to designated targets and facilitating highly specific molecular imaging. This advancement significantly improves the sensitivity and accuracy of US diagnostics, and numerous preclinical and clinical studies are currently exploring its broad range of applications (Table 1).

**Table 1:** Transforming ultrasound molecular imaging detection of cancers

Binding Ligands	Type	Diameter(nm)	Detection
carbonic anhydrase IX(CAIX)	nanobubble	478 ± 68	various malignant tumors [122]
prostate-specific membrane antigen	nanobubble	274 ± 8	prostate cancer [33]
SRC homology-2(SHP2)	nanobubble	535 ± 14	thyroid cancer [123]
nucleolin (NCL)	Nanobubble	459 ± 37	triple-negative breast cancer [124]
poly(ethylene glycol)(PEG)	gas vesicle	400 − 500	Lewis lung carcinoma [34]
poly(ethylene glycol) (PEG) and hyaluronic acid (HA)	gas vesicle	400 − 500	tumor detection [35]
VEGFR2	microbubble	/	anti-VEGF antibody treatment [125]
chitosan	nanodroplet	519 ± 72	imaging and treatment [126]
organic anion transporting polypeptides(OATPs)	nanodroplet	171 ± 57	cutaneous malignant melanoma [127]
αvβ3 integrin	microbubble	/	ovarian cancer using laying hens [128]
VEGFR2	microbubble	/	ovarian cancer [129]

**Fig. 2:**
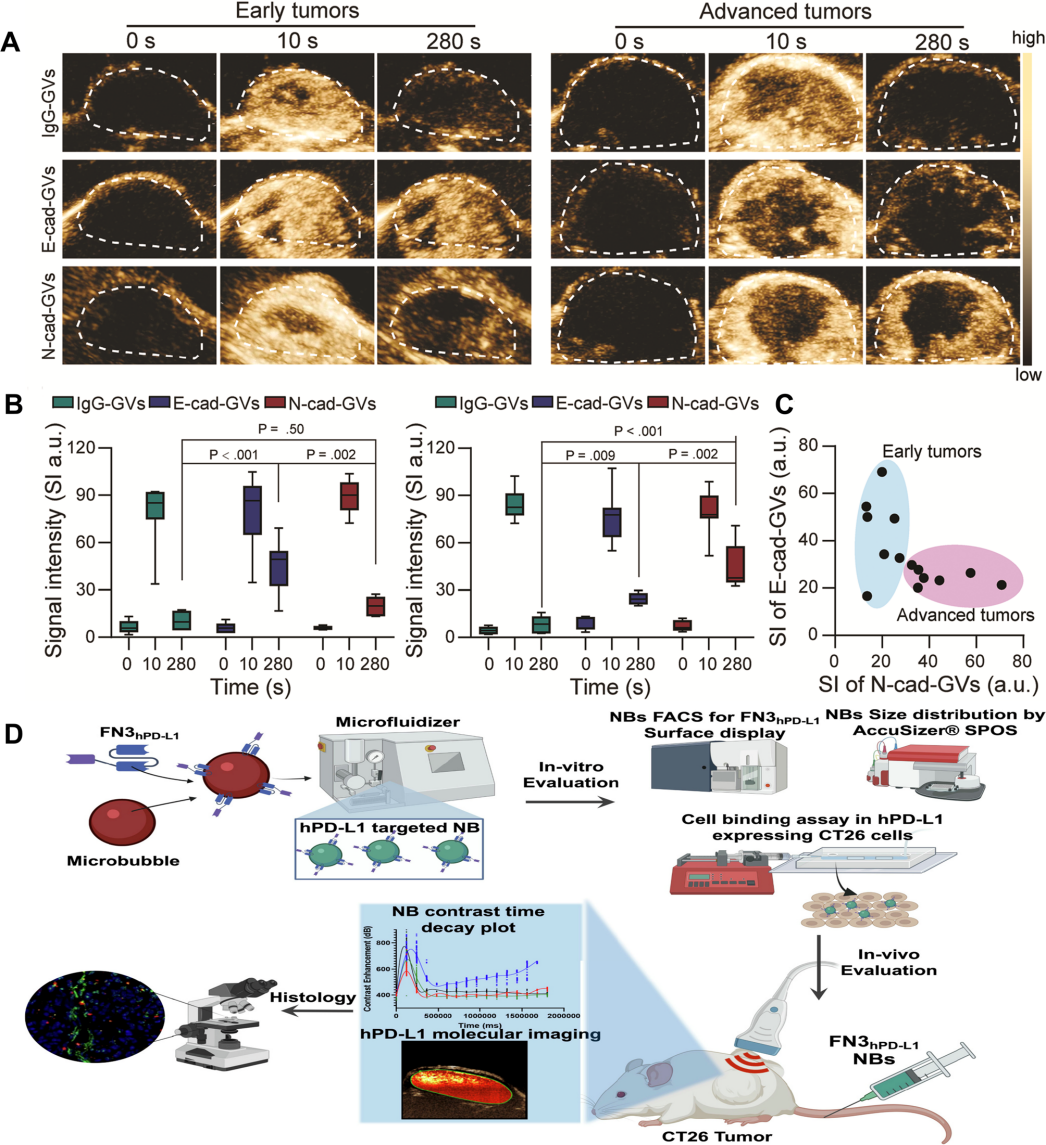
Ultrasound molecular imaging in tumor management applications. (**A**) To evaluate E-cadherin (E-cad) and N-cadherin (N-cad) expression in vivo, early- and late-stage tumor-bearing mice were injected with E-cad-GVs, N-cad-GVs, or control IgG-GVs. In early-stage tumors, IgG-GVs and N-cad-GVs showed rapid signal decay. At the same time, E-cad-GVs maintained significantly higher contrast intensity at 280 s.(**B**-**C**) E-cad-GVs produced stronger signals in early-stage tumors. In contrast, N-cad-GVs exhibited enhanced retention in late-stage disease. These results suggest that ultrasound molecular imaging (UMI) with cadherin-targeted GVs can dynamically track E-cad/N-cad shifts during tumor progression. Reproduced with permission from Ref [38]. Copyright© 2023 Wiley-VCH GmbH. (**D**) The investigations of hPD-L1 targeted FN3h_PD − L1_-NBs for ultrasonic imaging. Schematics of the preparation process and imaging performance of FN3h_PD − L1_-NBs via microfluidics-based reconstruction. Reproduced with permission from Ref [39]. Copyright © 2022 Elsevier Ltd

Certain molecules are overexpressed in tumors and can serve as targets for quantifying US contrast signals. As a result, molecular US imaging can detect signal changes before any visible morphological alterations occur in the tumor. This approach also allows for the evaluation of treatment responses to anti-angiogenic therapy, radiotherapy, and conventional chemotherapy. Vascular endothelial growth factor receptor 2 (VEGFR2), the primary receptor for VEGF, is highly expressed on the surface of neovascular endothelial cells within tumors. Its expression is closely associated with tumor prognosis and metastatic potential. Smeenge et al. [30] were the first to demonstrate contrast enhancement in prostate lesions using BR55, an US molecular contrast agent targeting VEGFR2. The feasibility and safety of BR55 were later confirmed in clinical studies for breast cancer [31] and liver cancer [32]. Additionally, Wang et al. [33]reported that prostate-specific membrane antigen (PSMA)-targeted nanobubbles could enhance the extravasation and retention of PSMA-expressing tumors. Other researchers have shown that nanobubbles coated with polyethylene glycol (PEG) or a combination of PEG and hyaluronic acid (HA) can evade the reticuloendothelial system (RES) and penetrate tumor vasculature via the enhanced permeability and retention (EPR) effect [34, 35]. Furthermore, epithelial-mesenchymal transition (EMT), a key driver of tumor metastasis, is characterized by decreased expression of E-cadherin and increased expression of N-cadherin [36, 37]. Targeted gas vesicles (GVs) directed at E-cadherin and N-cadherin (E-cad-GVs and N-cad-GVs) have been employed to assess EMT status and tumor metastatic potential [38] (Fig. 2A–C). These GVs can cross vascular barriers, specifically bind to cancer cells, and produce strong contrast imaging signals, demonstrating excellent tumor-targeting capabilities and offering a promising strategy for early detection of metastatic lesions. In parallel, programmed death-ligand 1 (PD-L1) is expressed in a variety of tumors, including melanoma, non-small cell lung cancer, Merkel cell carcinoma, breast cancer, and squamous cell carcinoma. The PD-1/PD-L1 axis suppresses T-cell activity in the tumor microenvironment, allowing tumors to escape immune surveillance. To target this pathway, Kumar et al. [39] developed PD-L1-targeted nanobubbles (designated PD-L1 FN3hPD-L1-NBs), which were covalently conjugated with FN3hPD-L1 nanobodies specific to human PD-L1 (Fig. 2D). These nanobubbles enable in vivo assessment of PD-L1 expression in the tumor microenvironment and facilitate US imaging of hPD-L1 expression. In CT26 mouse xenograft models, FN3hPD-L1-NBs produced approximately threefold higher CEUS signals compared to non-targeted nanobubbles. Histological analysis of tumor sections stained with hematoxylin and eosin revealed no significant tissue damage, supporting their biocompatibility. These nanobubbles offer valuable diagnostic insight into PD-L1 expression levels, contributing to more accurate prognostic evaluation and personalized treatment planning for tumor patients.

US imaging, due to its non-invasive nature and real-time capabilities, has become an essential tool for tumor screening. Conventional US generates images based on the reflection of sound waves, while CEUS employs MBs to amplify blood flow signals, thereby improving the visualization of tumor microvasculature. US molecular imaging extends these capabilities by enabling early diagnosis and therapeutic monitoring at the molecular level through the use of targeted nanobubbles that bind specifically to tumor biomarkers such as VEGFR2 and PD-L1. Additionally, multimodal ultrasonography integrates multiple imaging modalities, further enhancing diagnostic accuracy. Collectively, these advancements demonstrate significant potential in facilitating early tumor detection, evaluating treatment efficacy, and assessing patient prognosis.

## Application of US in tumor treatment

The fundamental principle of US therapy involves the use of sound waves to induce thermal and non-thermal effects within tissues without causing damage to the surrounding structures along the beam path [40]. The primary objectives are either to ablate pathological tissues or to stimulate tissue regeneration, thereby slowing disease progression [41]. While US has found applications across various medical disciplines, its role in oncology is particularly significant. Modern strategies for treating malignant tumors include surgery, radiotherapy, chemotherapy, and immunotherapy, with chemotherapy being the most commonly employed systemic treatment. However, even the most advanced chemotherapeutic regimens often fail to achieve the desired outcomes due to obstacles such as inefficient drug delivery, tumor heterogeneity, and the development of drug resistance. In this context, US is emerging as a versatile and promising tool in cancer therapy, serving both as a stand-alone therapeutic modality and as an adjunct to enhance the efficacy of existing treatments. For instance, US-mediated hyperthermia, particularly high-intensity focused US (HIFU), can selectively heat and destroy tumor cells while sparing adjacent healthy tissues. Moreover, US-assisted drug delivery has shown potential in improving therapeutic outcomes by increasing drug permeability and accumulation in target tissues. With ongoing technological advancements, the clinical applications of US therapy continue to expand, offering new avenues for optimizing cancer treatment [42].

### Thermal therapy

**Fig. 3:**
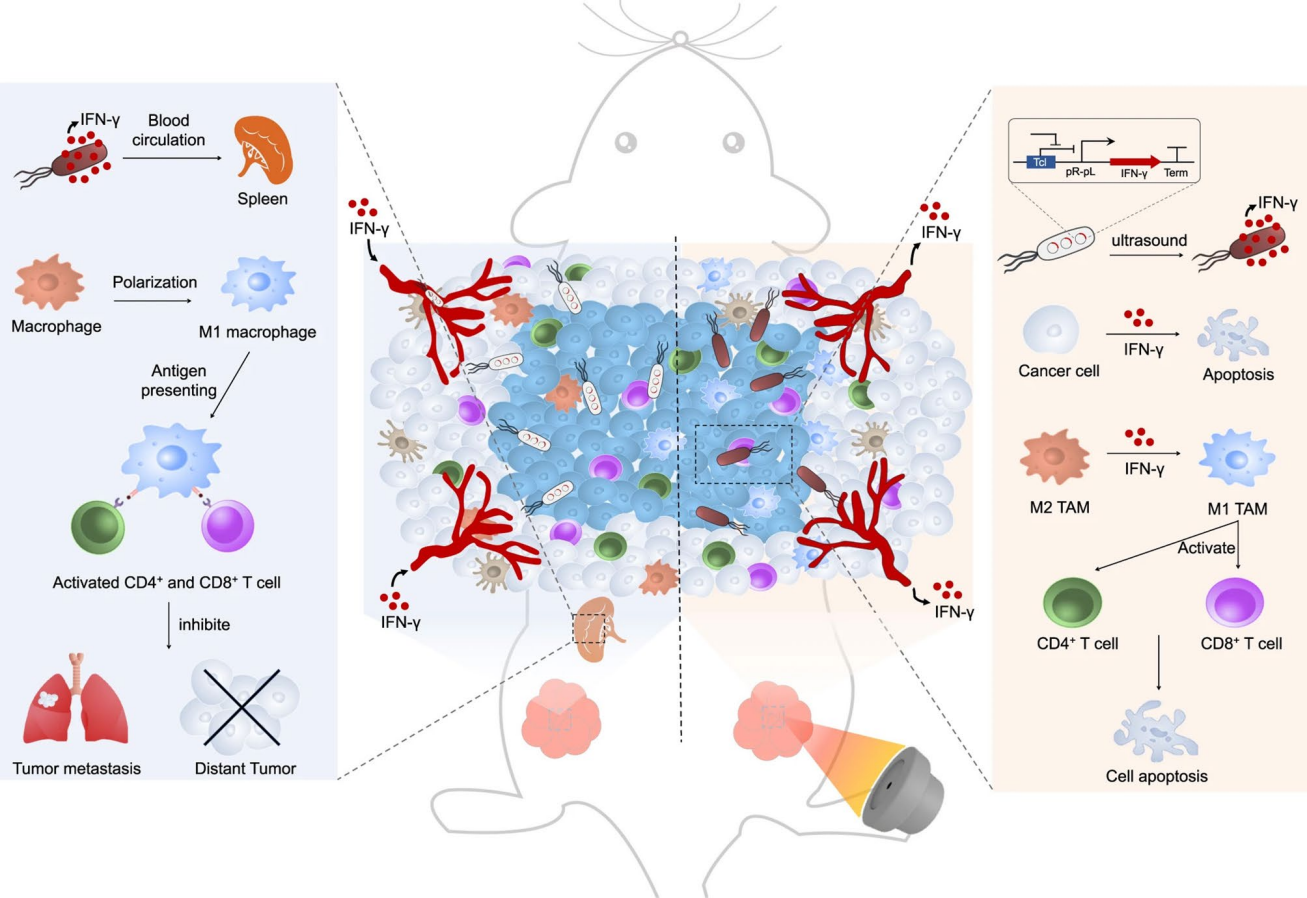
FUS-triggered local heating (42–45 °C) activates engineered ultrasound-responsive bacteria (URB) containing a thermosensitive IFN-γ gene circuit. This controlled IFN-γ expression induces three key antitumor effects: (1) direct cancer cell apoptosis, (2) macrophage repolarization from M2 to M1 phenotype, and (3) activation of CD4+/CD8 + T cells. The systemic IFN-γ response further enhances immune activation in the spleen, generating immunological memory that inhibits tumor metastasis. Reproduced with permission from Ref [54]. Copyright© 2023 Wiley-VCH GmbH

The thermal effect of US therapy arises from the absorption of US energy by target tissues, resulting in localized heating. Biological tissues possess specific acoustic absorption properties that enable a portion of the incident US energy to be converted into thermal energy, thereby increasing tissue temperature. The degree of temperature elevation depends on the intensity, frequency, and duration of US exposure. Under constant sound intensity, tissue temperature rises proportionally with exposure time until a point is reached where the increase plateaus due to thermal conduction. Once the tissue temperature stabilizes, further increases are mitigated as heat diffuses to surrounding areas. This results in a non-uniform temperature distribution, especially when US is focused locally or when the acoustic absorption properties of tissues vary. As the temperature gradient increases, thermal conduction becomes more pronounced until thermal equilibrium is achieved. At energy doses exceeding 55 °C, tissues undergo coagulative necrosis, resulting in irreversible cell death. The thermal effects of HIFU were first recognized in 1932, and its therapeutic potential was proposed soon after [40]. HIFU can precisely deliver ultrasonic energy to targeted lesions, generating localized hyperthermia that destroys tumor tissues without damaging adjacent healthy structures [43]. Interest in HIFU grew substantially in the 1960s, when Fry used it to create cortical lesions in an effort to slow the progression of Parkinson’s disease and other movement disorders [44]. By the late 20th century, HIFU had been adopted as a selective treatment modality in ophthalmology and neurosurgery. The development of MRI in the 1980s further revitalized interest in HIFU by enabling precise spatial guidance and the introduction of MR thermometry for real-time temperature monitoring [45]. A major milestone was reached in 2003 with the introduction of the first MR-guided focused US system (MRgFUS), laying the foundation for HIFU to become a widely accepted therapeutic modality [46]. In recent years, HIFU has been used to treat a range of both benign and malignant solid tumors. Unlike traditional cancer treatments such as chemotherapy, radiotherapy, and surgery, HIFU is entirely non-invasive, extracorporeal, and non-ionizing, making it uniquely suitable for treating both primary and metastatic solid tumors. Over the past decade, clinical trials using transrectal HIFU for prostate cancer have demonstrated promising results at more than 100 centers worldwide across Europe, the United States, and Asia. Follow-up studies conducted 2 to 5 years post-treatment have shown consistently low prostate-specific antigen (PSA) levels, with negative biopsy rates ranging from 60 to 90% [47, 48]. Furthermore, clinical applications of HIFU have improved prostate cancer control rates from 50% at 8 months to approximately 90% in recent trials [49]. In addition to focal therapy, whole-gland HIFU ablation has reduced tumor incidence by 17–35% and tumor volume by over 90% [50]. HIFU is especially promising for treating prostate cancer in patients who are obese, over 65 years of age, or ineligible for surgery [51]. For pancreatic cancer—a disease often diagnosed at an advanced stage with a 5-year survival rate below 5%—HIFU has emerged as a viable treatment option. It is applied as a standalone therapy, in combination with chemotherapy (e.g., gemcitabine), or as an adjunct after the failure of chemotherapy or radiotherapy [52]. Early findings from HIFU treatment have shown encouraging results, including significant tumor volume reduction and pain relief in up to 80% of patients. In studies involving 30 to 223 patients, average survival reached 12.5 months (ranging from 8 months to over 3 years), with a 50% tumor reduction rate for HIFU alone and overall response rates of 43.6% and 14.6% when combined with chemotherapy [53]. Moreover, while tumor ablation typically requires US frequencies ranging from 1 to 7 MHz, milder acoustic parameters (e.g., 960 Hz) can be used for US-controlled genetic modulation [54] (Fig. 3). One innovative approach involves combining interferon-gamma (IFN-γ) genes with temperature-sensitive therapeutic plasmids to engineer US-responsive bacteria (URB) capable of secreting IFN-γ during HIFU-mediated thermal therapy. This technique has been shown to activate IFN-γ expression through US-induced heating, thereby eliciting anti-tumor immune responses that inhibit tumor growth and metastasis.

### Sonodynamic therapy (SDT)

**Fig. 4:**
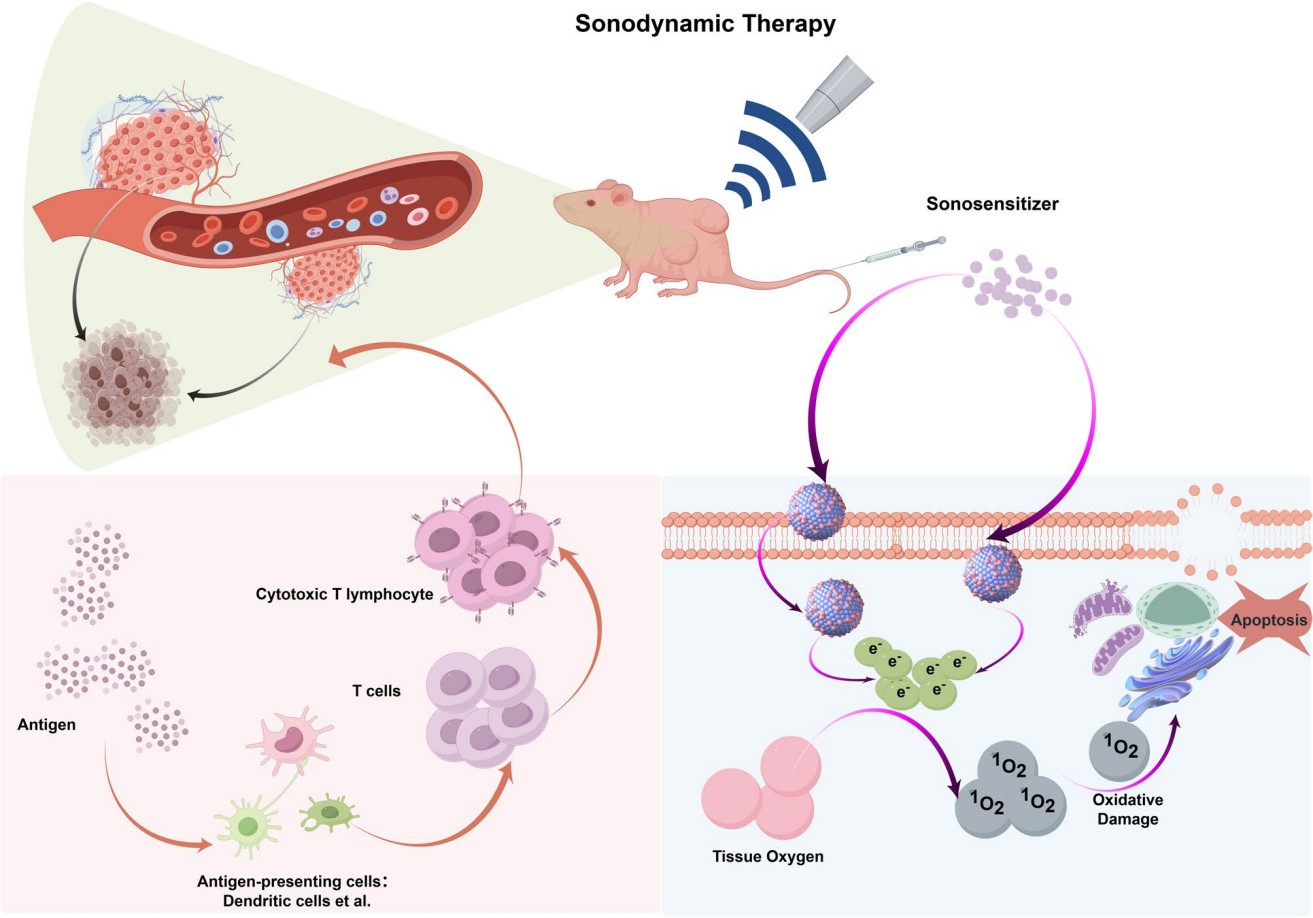
Schematic diagram of sonodynamic therapy. Ultrasound-triggered inertial cavitation (UIC) increases the production of ROS, induces antigen exposure and presentation, enhances DC maturation, and promotes more activated effector T cell infiltration, thereby inhibiting metastatic tumor cells (left pink area); The ultrasound action activates the photosensitizer, which in turn produces ROS, leading to apoptosis and necrosis of tumor cells (right blue area)

FUS is categorized into HIFU and low-intensity focused US (LIFU) based on varying sound frequencies. Theoretically, LIFU offers many of the benefits of HIFU while reducing the risk of tissue overheating associated with higher frequencies, thus improving treatment safety. In 1989, Yumita et al. introduced a therapeutic strategy combining low-intensity US with sonosensitizers for SDT [55]. When low-intensity US irradiates MBs in bodily fluids or soft tissues—either pre-existing or formed during US exposure—these bubbles can expand under low combined sound and static pressure, and contract when the pressure is high. This results in a breathing-like vibration or pulsation of the bubbles, typically classified as either stable (non-destructive) or transient cavitation. At lower intensities, cavitation remains steady and produces minimal destructive force, whereas increased sound intensity induces nonlinear bubble oscillation, collapse, and shockwave generation near the bubble surface [56]. This nonlinear stable cavitation can generate mechanical shear and localized microstreaming in surrounding tissues, disrupting cell membranes [57]. When the US intensity exceeds a specific pressure threshold, the bubbles rapidly expand past resonance size and implode violently [58], producing extreme local temperatures (up to 10,000 K) and pressures (81 MPa) [59, 60]. These extreme conditions elicit potent biological effects, including the production of reactive oxygen species (ROS), which induce tumor cell apoptosis and necrosis [61, 62]. Furthermore, ROS can trigger immunogenic cell death (ICD), activating adaptive immune responses [63]. The addition of sonosensitizers further lowers the cavitation threshold and promotes sonochemical reactions, enhancing ROS production and amplifying treatment efficacy [64] (Fig. 4). While SDT offers a non-invasive means to eliminate localized solid tumors, its systemic anti-tumor effects remain limited, lacking strong anti-metastatic potential. Therefore, the efficacy of SDT heavily depends on sonosensitizers, which are generally classified into organic and inorganic types [65]. Organic sonosensitizers include porphyrins, phthalocyanines, and their derivatives [66], while inorganic types encompass metal oxides (e.g., Ag_2_O, TiO_2_) and piezoelectric materials like black phosphorus and barium titanate [67, 68]. Organic sonosensitizers were pioneers in SDT applications [69–71]. For example, Wang et al. extracted a chlorophyll derivative (CHC) from spirulina, structurally identical to chlorin e6 (Ce6), and modified it with three substituents to produce DYSP-C34, which showed tumor-targeting and US-triggered ROS generation abilities [72]. After treatment with DYSP-C34 combined with US, the tumor area in liver tissue was reduced to just 2.2%, compared to 78.6% in the control group, demonstrating effective tumor suppression. Inorganic sonosensitizers also exhibit high SDT potential due to their robust physicochemical properties and multifunctionality. Ding et al. first demonstrated the potent sonodynamic activity of nitrogen-doped graphene quantum dots (N-GQDs) [73], which generated 3–5 times more ROS under US than conventional sonosensitizers. Thanks to the stability of pyrrole N and pyridine N within their graphene lattice, N-GQDs retained high sonodynamic efficiency even after tumor-targeting functionalization with folic acid (FA-N-GQDs). These functionalized N-GQDs produced abundant ROS upon US stimulation, activating oxidative stress responses in tumor cells via the PEX-p53 pathway, resulting in apoptosis rates as high as 95%. In murine subcutaneous tumor models, FA-N-GQDs rapidly and selectively accumulated in tumor tissues, and after two US treatments over 14 days, tumor volume was reduced by over 95%. Recent advances in nanotechnology have introduced new strategies for enhancing cancer therapy [62]. For example, Zhang et al. synthesized a multifunctional cascade nanoreactor to improve colon cancer SDT by simultaneously boosting ROS production and inhibiting autophagy [74]. Their system incorporated chloroquine (an autophagy inhibitor) and Ce6 into hollow polydopamine nanocores pre-doped with platinum nanoparticles (CCP@HP), and functionalized the surface with homologous tumor cell membranes (CCP@HP@M), enabling precise tumor targeting and significantly improved therapeutic outcomes, offering a novel approach for the precision treatment of deep-seated tumors.

### US-Assisted drug delivery

Compared to normal tissue environments, the tumor microenvironment exhibits distinct structural and compositional features [75], such as high cell density, leaky tumor vasculature, elevated interstitial pressure, an abnormal extracellular matrix, and the absence of functional lymphatic drainage. These unique characteristics present major obstacles to the effective delivery of chemotherapeutic agents, necessitating the development of innovative drug delivery strategies that can transport therapeutic agents from the vascular system into the tumor interior. US-assisted drug delivery systems (US-DDS) have been explored across various diseases and are particularly valued for their spatial and temporal controllability. This precise control enables the integration of diagnostic and therapeutic functions, underscoring their considerable potential in oncology. As previously discussed, MBs and NBs play a key role in enhancing both the therapeutic efficacy and imaging contrast of US-based treatments. These vesicular structures can also serve as drug carriers, facilitating targeted delivery to specific tissues and cells. US-DDS can be classified into several drug delivery approaches that leverage either the biological effects of US-induced MB/NB destruction or the regulatory mechanisms triggered by US interaction with these bubbles.

#### Promoting drug uptake

The tumor microenvironment is often highly complex, posing a significant barrier to the effective penetration of conventional chemotherapeutic agents, thereby limiting treatment efficacy. US can induce the cavitation of MBs, generating mechanical effects such as shock waves, microstreams, and shear stress. These physical forces disrupt vascular walls and cell membranes, resulting in pore formation and the loosening of tumor cell junctions, which enhances membrane permeability and facilitates the deeper penetration and accumulation of chemotherapeutic drugs within the tumor tissue [76]. Gourevich et al. utilized MRgFUS to evaluate doxorubicin uptake by MCF-7 cells, both with and without the presence of US and MBs [77]. Their findings showed a 3.2-fold increase in cellular drug uptake under stable nonlinear cavitation. MRgFUS not only offers a novel technique for quantifying cavitation dosage but also significantly advances the clinical translation of US combined with MBs as a non-invasive and targeted strategy to enhance anticancer drug delivery [78]. Similarly, Bressand et al. demonstrated that US-mediated microbubble cavitation effectively enhanced the targeted delivery of paclitaxel to pancreatic tumors, significantly reducing tumor volume in a subcutaneous pancreatic cancer mouse model, while simultaneously lowering drug dosage and minimizing side effects [79, 80]. Michon et al. also reported that US-targeted MBs increased blood flow in skeletal muscle by amplifying nitric oxide (NO) signaling in endothelial cells, an effect that was further potentiated with the addition of sodium nitroprusside—offering promising implications for improving radiotherapy in solid tumors [81].

#### Drug loading

**Fig. 5:**
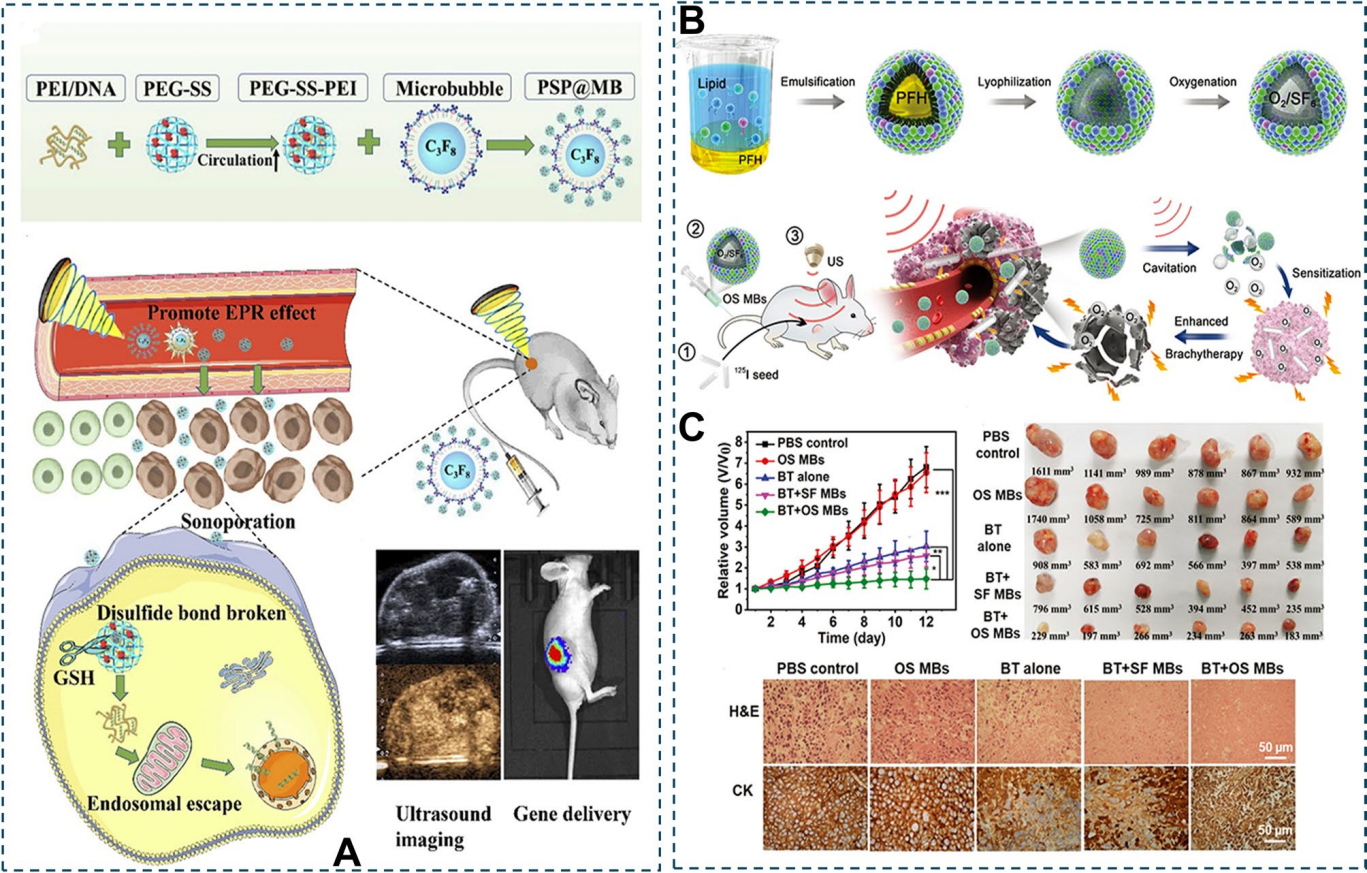
Ultrasound-mediated effective drug delivery. (**A**) The novel PSP@MB and ultrasound-mediated gene delivery system could efficiently target cancer stem cells. Reproduced with permission from Ref [90]. Copyright© 2019 Dove Medical Press Ltd. (**B**, **C**) Through ^125^I seed implantation, intravenously administered OS MBs are ultrasound-triggered to induce oxygen release, thereby alleviating local hypoxia in solid tumors and enhancing the efficacy of brachytherapy.Reproduced with permission from Ref [98]. Copyright© 2019Wiley-VCH GmbH. Notes: PEI, polyethylenimine; PEG, Polyethylene glycol; SS, disulfide bond; MB, microbubble; EPR, enhanced permeability and retention effect; GSH, glutathione; OS MBs, O_2_/SF_6_ microbubbles

Systemic circulation of drugs often results in low local drug concentrations at target sites, limiting therapeutic efficacy due to insufficient accumulation at lesion sites [82]. This limitation can be addressed by encapsulating drugs within MBs or by attaching them covalently or non-covalently to MB surfaces. For example, Chen et al. developed cisplatin-loaded MBs that showed enhanced antitumor effects under US exposure and reduced cisplatin accumulation in the kidneys and liver [83]. Liang et al. designed amphiphilic Janus camptothecin-fluorouracil (CF) lipid MBs with a drug loading efficiency of 56.7 ± 2.3%, demonstrating a 14-fold increase in controlled drug release under US compared to passive release, resulting in a tumor inhibition rate of 72.4% for CF-MBs + US versus 21.6% for CF liposomes [84]. However, the gap sizes in newly formed tumor vasculature (380–780 nm) limit the extravasation of conventional US contrast agents (1–5 μm). Polymer MBs can shrink to approximately 400 nm under US, retaining acoustic responsiveness and enabling deeper penetration into tumor stroma for enhanced post-release drug retention and diffusion [86]. Since single-drug efficacy may be inadequate, dual-drug loading strategies have been explored. For instance, gemcitabine (hydrophilic) can be surface-bound via biotin affinity, while paclitaxel (hydrophobic) is embedded in the MB core; combined US application led to marked tumor reduction [85]. Beyond chemotherapeutics, MBs can be functionalized with antibodies, growth factors, DNA, or RNA through covalent binding, biotin-avidin interaction, or electrostatic adsorption ctors, DNA, and RNA via covalent bonding, biotin affinity, or electrostatic absorption [86]. US-targeted microbubble destruction (UTMD) significantly enhances gene transfection. Rychak et al. showed that siRNA-loaded MBs targeting the PTEN tumor suppressor doubled knockdown efficiency under US compared to free siRNA [87]. UTMD also boosts chemotherapy by aiding miRNA transfection [88]or regulating protein expression in tumor-related pathways [89]. CHUN et al. conjugated PEG-SS-polyethylenimine (PSP) to MB surfaces via biotin-avidin bonds, creating PSP@MBs that, under US exposure, improved gene delivery to solid tumors while reducing off-target toxicity (Fig. 5A) [90]. Elevated tumor interstitial pressure often hampers the transport of large molecules like antibodies; UTMD generates “sono-pores” in the vascular endothelium, improving their penetration into tumor tissue [90]. For instance, MBs conjugated with NF-κB antibodies have been used for US imaging of inflammatory bowel disease [91]. Thomas et al. developed EGFR-targeted lipid MBs combined with US cavitation for radiolabeled chemotherapy, encapsulating doxorubicin in liposomes functionalized with Indium-111-labeled EGF; this strategy significantly enhanced drug uptake in MDA-MB-468 xenografts, even with poor tumor angiogenesis [92]. Surface modification of drug-loaded MBs can also enable targeted delivery. Yuan et al. engineered MBs conjugated with anti-ICAM-1 antibodies and Endostar to target plaque neovascularization, achieving efficient drug delivery upon US stimulation [93]. Overall, MBs can be conjugated with a variety of small and large biomolecules to enhance intracellular drug delivery under US stimulation.

**Fig. 6:**
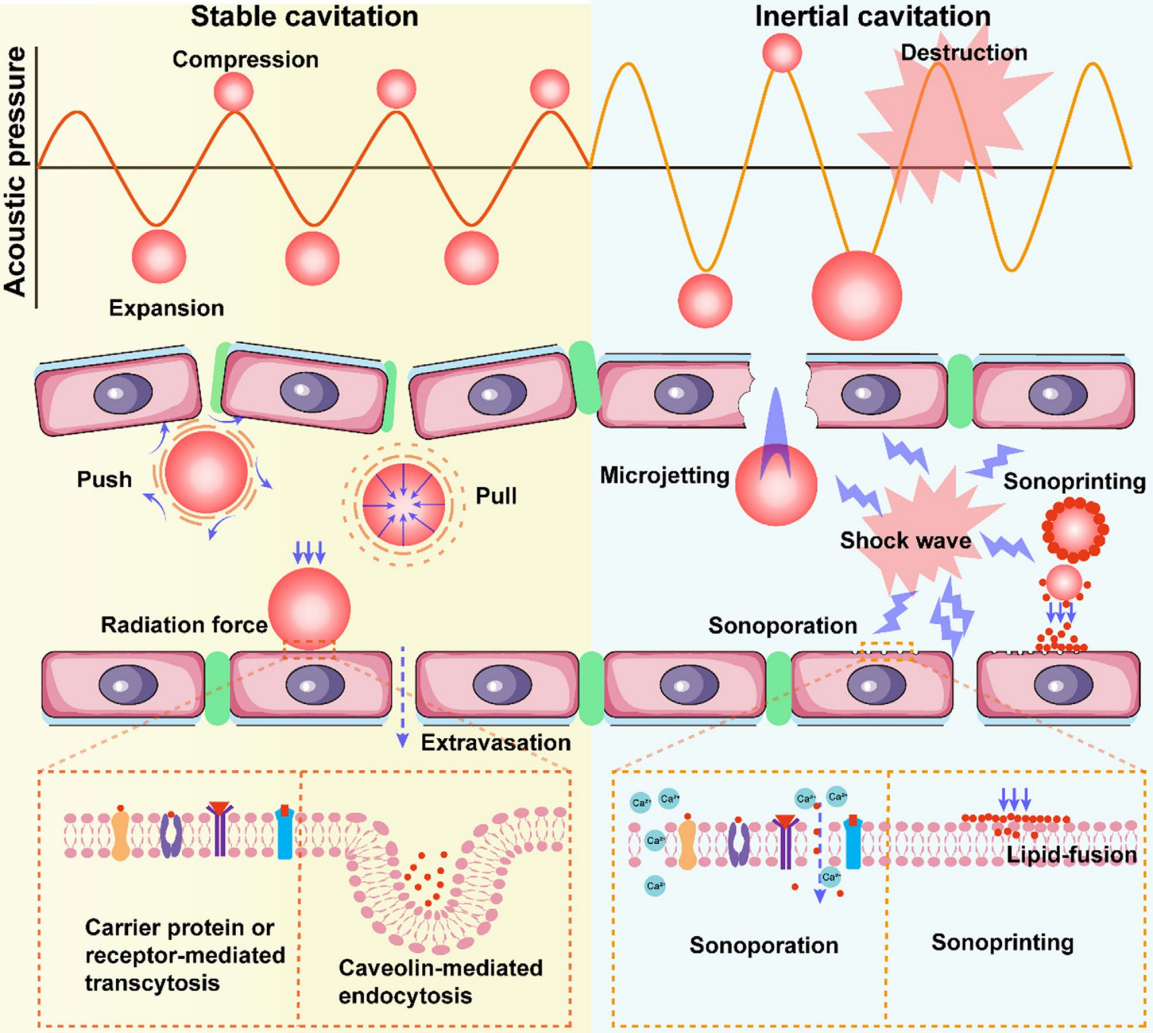
Schematic diagram of the biophysical effects of ultrasonic waves on MBs. In the stable cavitation phase, FUS upregulates carrier protein-receptor mediated transcytosis and caveolin-mediated endocytosis, and downregulates tight junction protein-mediated BBB opening (left yellow diagram). In the inertial cavitation phase, strong shock waves, microflows, micro-jets, and tangential stresses generated by the collapse of MBs lead to cell membrane perforation and large-scale blood-brain barrier opening (right blue diagram).Reproduced with permission from Ref [121]. Copyright © 2022 Elsevier Ltd

Furthermore, gas molecules such as NO, hydrogen sulfide (H_2_S), carbon monoxide (CO), and oxygen (O_2_) play crucial roles in cellular signal transduction and have demonstrated significant therapeutic potential in radiotherapy, chemotherapy, photodynamic therapy, SDT, and immunotherapy [69]. Due to their small molecular size, these gases can diffuse more efficiently across vascular endothelial cells and the blood-brain barrier (BBB) than conventional drugs. Moreover, gas molecules typically exhibit lower toxicity and higher permeability and accumulation compared to traditional pharmacological agents [94]. As a result, US-assisted delivery of gas molecules has garnered increasing attention. A major challenge in solid tumor treatment is the hypoxic tumor microenvironment, which contributes to hypoxia-induced radioresistance and reduces therapeutic efficacy. In conventional radiochemotherapy, O_2_ is often employed as a radiosensitizer to enhance treatment outcomes. Vaidya et al. developed MBs composed of D-α-Tocopherol PEG 1000 succinate (TPGS) and sorbitan monostearate, which improved MB stability and achieved a high O_2_-loading efficiency of up to 10.49%, enabling sustained O_2_ delivery [95]. Additionally, the composition of the MB shell and the O_2_ content within the gas core significantly influence the stability of lipid-based MBs. Using phospholipids with longer hydrocarbon chains or reducing the O_2_ fraction by incorporating gas mixtures can markedly extend MB half-life [96], thereby improving O_2_ delivery to tumor vasculature under US activation. Peng et al. employed US-triggered O_2_-carrying MBs to deliver O_2_ directly to hypoxic tumor regions, significantly enhancing the efficacy of localized radiotherapy in nasopharyngeal carcinoma models [97]. In a breast cancer study, tumors treated with O_2_-loaded MBs followed by radiotherapy showed a tumor volume increase of only 41%±1%, compared to a dramatic 337%±214% increase in the group receiving radiotherapy alone (Fig. 5B, C) [98]. These findings underscore the potential of US-mediated gas delivery to overcome hypoxia-induced resistance and improve therapeutic outcomes in solid tumors.

### Opening biological barriers

The BBB constitutes a critical physiological barrier that restricts the entry of therapeutic agents into the central nervous system (CNS), thereby posing a major challenge to the treatment of neurological disorders and brain tumors [99]. FUS has emerged as a promising, non-invasive technique to transiently and reversibly open the BBB, allowing for the targeted delivery of a wide range of therapeutic agents. These include small-molecule drugs [100], cells [101], viral vectors for gene therapy [94], and immunotherapeutic agents [102]. The feasibility and safety of transcranial FUS for BBB disruption have been validated in preclinical studies, including non-human primate models, demonstrating its potential for clinical translation.

The US threshold required to open the BBB can be reduced by up to 100-fold through the activation of MBs [103]. When exposed to US, MBs undergo expansion and contraction depending on the frequency of the US waves, generating mechanical forces, microstreaming, and acoustic radiation that facilitate their interaction with the vascular endothelium. This mechanical activity enables the transient disruption of the BBB, thereby permitting drug molecules to cross into the brain parenchyma [104] (Fig. 6). Lipsman et al. demonstrated the safe, reversible, and repeatable non-invasive opening of the in patients using low-frequency US in combination with MBs, with full restoration of BBB integrity occurring within 24 h [105]. Typically, MBs are administered intravenously alongside therapeutic agents, and focused US locally enhances endothelial permeability, improving drug uptake by target brain regions. In 2022, Ye et al. [106]introduced an innovative strategy combining US and MBs with intranasal delivery. This approach leverages the nasal route to bypass the BBB, minimizing systemic exposure and associated risks, while focused US-induced MB cavitation enhances the transport of intranasally administered drugs to specific pathological sites within the brain. Both intravenous and intranasal methods utilizing US and MBs significantly improve drug delivery efficiency to CNS regions that are otherwise inaccessible to conventional therapeutics, offering promising avenues for treating neurological disorders. Furthermore, the rapid development of smaller NBs with improved stability and enhanced echogenicity has addressed the limitations of MBs, such as their relatively large size and limited drug loading capacity. NBs enable more efficient extravasation into surrounding tissues and facilitate US-triggered, targeted drug release [107].

### Activating immunity

US-mediated immunomodulation represents an emerging field within US therapy, with increasing evidence that the benefits of FUS extend beyond localized treatment. FUS can exert direct effects on tissue and enhance the delivery of immune stimulants, thereby triggering immune responses with systemic implications for various diseases. Boiling histotripsy (BH), a technique that employs pulsed HIFU, generates high-amplitude shock waves and induces localized heating and shock-driven bubble activity at targeted lesions, resulting in tissue liquefaction [108]. This process not only facilitates thermal ablation of solid tumors but also promotes the release of danger-associated molecular patterns, including tumor antigens and other immunogenic factors, which in turn stimulate adaptive immune responses and enhance host antitumor immunity [109]. The therapeutic efficacy of this immune activation can be further amplified through the use of immunotherapy, such as immune checkpoint inhibitors [110, 111]. Immune checkpoints are regulatory molecules that inhibit cytotoxic T cell activity [114] or suppress innate immune responses [115], and checkpoint inhibitors function by blocking these pathways to reactivate endogenous immune responses. While checkpoint inhibitors have demonstrated promising outcomes in certain patients, their overall response rates in clinical trials remain limited, highlighting the need for combination strategies to improve therapeutic success [112]. US can potentiate the effects of checkpoint inhibitors by enhancing both innate and adaptive immune responses [113]. For instance, Singh et al. combined BH with in situ administration of anti-CD40 agonist antibodies (αCD40) to improve the efficacy of immune checkpoint blockade in a refractory mouse melanoma model. This combination stimulated strong intratumoral infiltration of immune cell populations and induced systemic responses at distant, untreated tumor sites, leading to the suppression of lung metastases and increased survival rates in tumor-bearing mice [110].

Histotripsy is an innovative, non-invasive, and non-thermal ablation technique that employs US-induced cavitation to mechanically disrupt tissues and release various immune-stimulating factors [114, 115]. Preclinical studies have shown that histotripsy activates the innate immune system in vivo, resulting in a significantly higher infiltration of CD8 + T cells compared to conventional treatments such as thermal ablation, radiotherapy, or radiofrequency ablation [116]. Additionally, US has been demonstrated to enhance the accumulation of immune checkpoint inhibitors within tumors, particularly when used in conjunction with MBs that target the vasculature and increase vascular permeability. This strategy enables the efficient and localized delivery of checkpoint inhibitors or immune adjuvants by loading them onto MBs, thereby improving therapeutic efficacy while reducing systemic toxicity [117, 118]. Bulner et al. reported that the combination of US MBs and checkpoint inhibitors produced superior antitumor effects compared to monotherapy, leading to robust antitumor responses and prolonged survival in murine models [119]. In the CNS, the combined application of US and MBs facilitates modulation of the BBB, triggering acute sterile inflammatory responses that are essential for developing immune-based therapies while maintaining treatment safety [120].

With ongoing technological advancements, US therapy is gaining increasing traction in cancer treatment, with HIFU showing promising results in managing conditions such as prostate and pancreatic cancers. SDT also demonstrates considerable potential in treating localized solid tumors by improving drug permeability and stimulating tumor-specific immune responses. Overall, US therapy represents a novel and effective adjunctive approach to conventional cancer treatments, particularly for localized tumors, offering significant application prospects. Nonetheless, several challenges persist, including limitations in deep tissue penetration, the stability of sonosensitizers, and the precise modulation of immune responses. Consequently, future research is increasingly geared toward refining multimodal synergistic therapies to overcome these barriers and maximize therapeutic outcomes.

## Prospects and challenges

US has emerged as a pivotal tool in the era of tumor precision medicine, offering promising diagnostic and therapeutic capabilities. However, translating its immense potential from laboratory and preclinical settings into routine clinical application remains fraught with critical challenges. Chief among these is the need for thorough validation of novel US contrast agents and sonosensitizers—especially those involving nanomaterials. Comprehensive, long-term clinical studies are essential to evaluate their biocompatibility, metabolic clearance, and potential for organ-specific or systemic toxicity. Furthermore, whether involving molecular probes, sonosensitizers, or multifunctional theranostic systems, detailed pharmacokinetic and pharmacodynamic profiles must be established alongside robust assessments of long-term safety and therapeutic efficacy. In addition, the clinical value of these emerging technologies must be demonstrated clearly in comparison to existing diagnostic and treatment modalities. To ensure their integration into healthcare systems, standardized quantitative indicators must be developed to evaluate feasibility, cost-effectiveness, and sustainability. Addressing these translational challenges requires a concerted effort from academia, industry, and regulatory bodies to formulate unified technical standards and advance regulatory science. Such collaboration is vital for accelerating the safe, effective, and practical clinical translation of US-based diagnostic and therapeutic innovations.

In summary, US offers distinct advantages over radiation and magnetic fields, including non-invasiveness, cost-effectiveness, operational simplicity, and precise control, making it less restricted in clinical applications. Currently, US-based diagnostic and therapeutic systems are being applied across diverse disease models, including CNS disorders, cardiovascular diseases, musculoskeletal conditions, and various cancers. US molecular imaging using contrast agents has expanded the scope of traditional US by enabling disease diagnosis and characterization at the molecular level, as demonstrated in numerous animal and preclinical studies. Targeted US contrast agents can detect metabolic changes and have proven effective in early tumor detection, treatment monitoring, and image-guided therapy, thereby enhancing visualization and the quality of care. However, factors such as operator technique, equipment limitations, and patient variability continue to affect the sensitivity and specificity of CEUS, necessitating extensive clinical evaluation and collaboration between academia and industry to facilitate clinical translation. Advances in molecular chemistry, US physics, and imaging technologies are expected to further enhance the sensitivity and specificity of tumor US molecular imaging. Meanwhile, sonosensitizers have evolved through progress in materials science and nanotechnology, but clinical translation remains hindered by issues such as suboptimal pharmacokinetics, instability, insufficient targeting specificity, and potential toxicity. Although therapeutic US effects—such as thermal ablation, cavitation, and ROS generation—are increasingly understood, the complex, overlapping biological mechanisms remain inadequately explored, limiting broader application. To fully realize the clinical potential of US, there is an urgent need to investigate its underlying mechanisms, refine sonosensitizer design, and optimize US parameters to establish a robust theoretical and practical foundation. Additionally, integrating US with emerging technologies—such as wearable systems, omics-based analysis, and artificial intelligence—could drive forward personalized and precise medical interventions. Future developments may enable capabilities like biomechanical sensing, remote US control, intelligent diagnostics, and programmable nanorobots, positioning US as a cornerstone in tumor precision medicine by seamlessly combining diagnostic imaging with therapeutic functionality.
